# Targeted herbicide spraying systems: role of nozzle type, number of nozzle activation, nozzle orientation, and boom height on spray coverage and weed control

**DOI:** 10.1002/ps.70629

**Published:** 2026-02-21

**Authors:** Zaim Ugljic, Ahmadreza Mobli, Ryan DeWerff, Rodrigo Werle

**Affiliations:** ^1^ Graduate Student, Department of Plant and Agroecosystem Sciences University of Wisconsin‐Madison Madison WI USA; ^2^ Scientist, Department of Plant and Agroecosystem Sciences University of Wisconsin‐Madison Madison WI USA; ^3^ Research Specialist, Department of Plant and Agroecosystem Sciences University of Wisconsin‐Madison Madison WI USA; ^4^ Associate Professor, Department of Plant and Agroecosystem Sciences University of Wisconsin‐Madison Madison WI USA

**Keywords:** herbicide efficacy, site‐specific herbicide application, smart sprayer, spot‐spraying, targeted herbicide application technologies

## Abstract

**Background:**

Differences in ground‐based sprayer setup within targeted herbicide application systems can influence spray coverage and herbicide efficacy. This research aimed to improve understanding of how these factors affect spray coverage and weed control using complementary field and controlled‐environment experiments.

**Results:**

Multiple TP40015E nozzles provided the greatest spray coverage, whereas a single DG80015 nozzle produced the lowest coverage. A boom height of 53 cm resulted in greater spray coverage (34%) than 76 cm (28%; Studies 1 and 2). Under simulated wind conditions (10 km h^−1^), TP40015E and DG80015 nozzles produced similar spray coverage (25%). A 53 cm boom height provided greater spray coverage under both no‐wind (52%) and wind (32%) conditions compared to 76 cm (41% no‐wind *versus* 18% with wind; Study 2). Multiple‐nozzle activation resulted in higher spray coverage than a single‐nozzle activation under no‐wind (58% *versus* 36%) and wind (29% *versus* 19%) conditions. Multiple nozzles also resulted in greater weed control (> 92%) and biomass reduction (95%) than single‐nozzle activation (78% control and 87% biomass reduction; Study 3). Under no‐wind conditions, conventional 0° and 30° rearward inclined nozzle orientations provided comparable spray coverage (≥ 42%) and did not differ in weed control or biomass reduction. In contrast, under wind conditions, the 30° rearward inclined orientation resulted in the lowest spray coverage (16%; Study 5).

**Conclusion:**

Regardless of boom height, nozzle orientation, and wind, activation of multiple nozzles resulted in better spray coverage and weed control than single nozzle activation. © 2026 The Author(s). *Pest Management Science* published by John Wiley & Sons Ltd on behalf of Society of Chemical Industry.

## Graphical abstract

Targeted herbicide application technology performance depends on sprayer configuration. Across five studies, activation of multiple, overlapping nozzles and maintaining appropriate boom height consistently improved spray coverage and weed control, highlighting the importance of optimized sprayer setup for effective and reliable weed control.

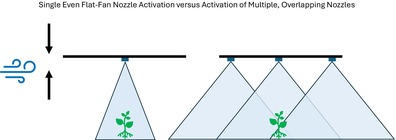

## 1. INTRODUCTION

Delivering the full herbicide dose to the targeted area is essential for optimizing efficacy and achieving effective weed control.^1^ Herbicide application is a complex process requiring careful attention to spray configuration parameters to ensure that spray droplets reach the intended target.^2^ Incorrect nozzle selection, non‐ideal boom height, and miscalibrated equipment can all negatively impact the accuracy of herbicide delivery,^3^ reduce pattern uniformity,^4^, ^5^ and spray coverage,^6^, ^7^ ultimately reducing weed control efficacy.^8^, ^9^, ^10^ Therefore, selecting an optimal sprayer configuration is of great importance to ensure precise herbicide delivery to the target.

Advancements in ground‐based sprayer technology over the past decade have led to the development of targeted herbicide application technologies (THATs) capable of site‐specific weed detection and treatment. These systems include applications used in fallow or preplant settings (green‐on‐brown) as well as real‐time weed detection within established crops (green‐on‐green).^11^, ^12^ These modern systems integrate camera‐based sensing with machine learning algorithms, often based on artificial intelligence, to distinguish weeds from crops and activate specific nozzle(s) to apply herbicide only where weeds are present, offering a more precise alternative to traditional broadcast applications.^13^, ^14^, ^15^, ^16^ Differences among THATs (also described as smart sprayers, targeted sprayers, optical sprayers, intelligent sprayers, spot‐sprayers, or precision sprayers in the literature) have been reported across manufacturers, particularly in the types of detection technologies employed (e.g., red‐green‐blue (RGB) or infrared cameras), recommended spray nozzle types (e.g., even flat‐fan (hereafter referred to as ‘even’) or overlapping tapered flat‐fan (hereafter ‘overlapping’)), nozzle activation configurations, which may involve either a single even or multiple overlapping nozzle activation, and nozzle orientation (e.g., conventional 0° straight down or 30° rearward inclined). For instance, John Deere's See & Spray™ system (Blue River Technology, Santa Clara, CA, USA) uses proprietary high‐resolution camera systems to detect weeds and activates multiple overlapping nozzles 38° rearward inclined.^16^, ^17^ In contrast, Greeneye Technology™ (Tel Aviv‐Yafo, Israel) uses proprietary high resolution cameras and activates a single even nozzle conventionally oriented (0°) upon weed detection, with the option to turn on one adjacent nozzle on each side,^18^ whereas One Smart Spray™ (Bosch BASF Smart Farming, Cologne, Germany) using RGB cameras with red/near infrared filter activates a single even nozzle conventionally (0°) oriented.^19^ These design variations can affect application precision by influencing spray coverage and herbicide efficacy.^20^

Targeted herbicide application technologies have been reported to provide weed control levels comparable to broadcast applications.^19^, ^21^, ^22^, ^23^ However, under variable field conditions, such as uneven terrain, fluctuating boom height, and changing wind speed, activation of a single even or multiple overlapping nozzles may result in insufficient delivery of herbicide to the target.^24^ The selection between even and overlapping nozzle types significantly influences spray characteristics, including droplet size distribution, coverage uniformity, and drift potential.^25^, ^26^, ^27^, ^28^ Furthermore, within each nozzle category, factors such as spray angle, operating pressure, nozzle orientation, and nozzle spacing further modulate spray performance and the risk of off‐target movement.^25^, ^26^, ^27^, ^28^ Overlapping nozzles, such as the Drift Guard (DG) (TeeJet, Spraying Systems Co., Wheaton, IL, USA), are intended for broadcast application and rely on 100% overlap (50% from each adjacent nozzle) to ensure uniform spray distribution.^18^, ^29^, ^31^ In contrast, even nozzles, such as the TeeJet Flat Spray (TP) (TeeJet, Spraying Systems Co.), are designed for banding applications and are typically used in targeted spraying for single nozzle activation systems, where uniform coverage across the entire width of the spray pattern is required.^30^, ^31^ Even nozzles are not designed to overlap and their band width determines the rate they deliver. As a result, precise nozzle spacing and boom height are critically important to ensure accurate spray rates with even nozzles. Boom stability is a critical factor in large‐scale herbicide applications, where uneven terrain, machine speed, and field conditions often lead to fluctuations in boom height.^17^ Such variations can disproportionately affect spray distribution, leading to under‐ or over‐application within the treated band.^24^

While data on droplet size for individual nozzle types are available,^31^ the specific effects of nozzle activation (single *versus* multiple), nozzle orientation (conventional 0° *versus* 30° rearward inclined), and boom height on spray coverage and weed control with THAT remain poorly understood and warrant further investigation. Understanding how nozzle and boom configurations influence spray coverage, particularly in the presence of wind, is critical for optimizing targeted herbicide applications, ensuring efficacy, and supporting the broader adoption of THATs. Such research can provide valuable guidance to technology manufacturers and end‐users across the United States and beyond, where adoption of this technology is expected to increase in the future, and will assist them in making informed decisions once the technology becomes widely available.^32^ Therefore, five studies were conducted to assess spray coverage and weed control simulating THAT. The experiments evaluated the effects of nozzle type (even *versus* overlapping), number of activated nozzles (simulation single (one) *versus* multiple (three) nozzle activation), nozzle orientation (conventional 0° *versus* 30° rearward inclined), and boom height (53 cm *versus* 76 cm) under field conditions. In addition, targeted herbicide applications were simulated under no wind and presence of wind in a controlled environment to further examine the effects of wind and sprayer configuration on spray coverage.

## 2. MATERIALS AND METHODS

### 2.1. Spray coverage assessment (Study 1 – field environment)

**Figure 1:**
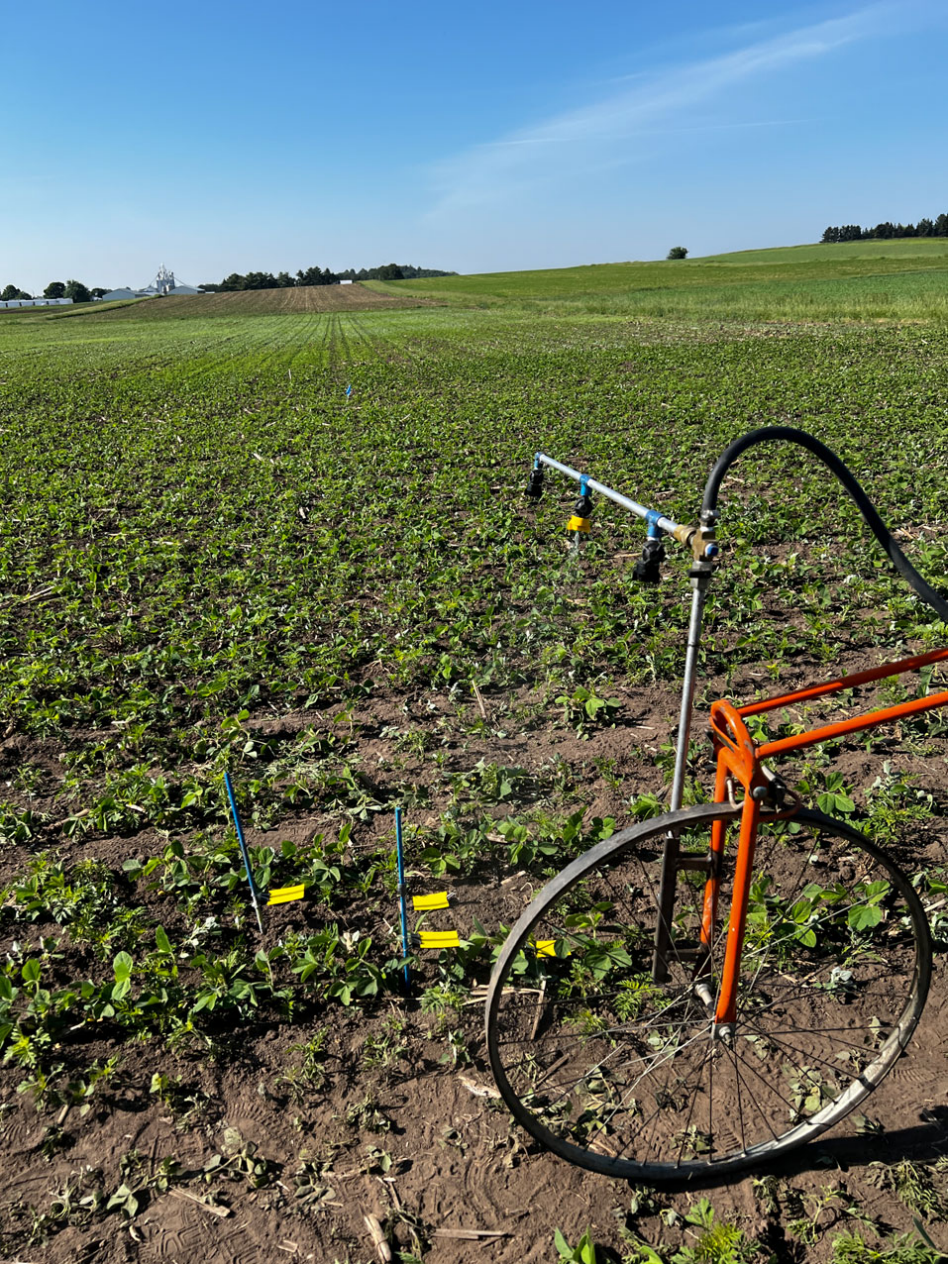
A hand‐held boom mounted on a bicycle wheel was used to apply treatments in all experiments conducted in Wisconsin during the 2023–2024.

Field experiments were conducted in 2023 and 2024 at the Arlington Agricultural Research Station (43.30° N, 89.34° W) near Arlington, WI, USA (Table 1), to evaluate spray coverage using a simulated THAT configured with different nozzles and boom height treatments. Experimental units consisted of plots measuring 9.1 m in length and 1.5 m in width, each containing four soybean rows spaced 38 cm apart. Soybean was planted at a density of 345 800 seeds ha^−1^ into a freshly tilled field. Treatments were arranged in a 2 × 2 × 2 factorial design within a randomized complete block design (RCBD) with four replicates. Factors included: nozzle type, even (TP40015E) *versus* overlapping (DG80015; TeeJet Spraying Systems Co.); number of nozzles, single *versus* multiple; and boom height, 53 cm *versus* 76 cm. Both nozzle types produced fine droplets under the operating conditions (pressure of 275 kPa)^31^ used in this study. Fine droplets were intentionally selected to serve as a proof‐of‐concept for evaluating spray coverage. A boom height of 53 cm was selected as ideal height for even nozzle treatment, as it allows for a theoretical spray coverage of 38 cm, corresponding to the row spacing in the soybean studies (Figure S1).^18^ A boom height of 76 cm was used to simulate a 25 cm vertical boom sway/movement, as observed by sprayer manufacturers/practitioners in field conditions.^17^ At this same boom height, the overlapping (DG80015) nozzle produced a substantially wider theoretical band width (89 cm), which increased to 128 cm at a 76 cm boom height which was likely to contribute to reduced spray coverage and lower effective dose delivery compared to multiple nozzle activation.^18^ Moreover, applicators may also decide to operate at suboptimal taller heights to prevent wide booms from contacting the ground, justifying the need for this treatment level. The boom height was measured from the nozzle tip to the target (water‐sensitive card (7.6 cm × 5.2 cm; Syngenta, Basel, Switzerland)). The application information is provided in Table 2. Applications were made as a broadcast spray using water with a carbon dioxide (CO_2_) pressurized hand boom calibrated to deliver 140 L ha^−1^ at 275 kPa with nozzles positioned 38 cm apart mounted on a bicycle wheel (Fig. 1). Nozzle flow rate, travel speed, and operating pressure were held constant across all treatments, and only boom height was varied; therefore, changes in effective spray width with increasing boom height resulted in corresponding changes in carrier volume per unit area. Spray volume was intentionally not adjusted for the higher boom height treatments to simulate real‐world field conditions, where application volume is typically calibrated based on an ideal boom height. As a result, under‐dosing was expected for even nozzle with elevated boom height treatment. Water‐sensitive cards (7.6 cm × 5.2 cm; Syngenta) were placed on wooden card holders (10 cm × 10 cm) at a height of 10 cm from the ground, approximating the height of weed canopies (common recommended weed height for foliar herbicide applications)^33^ in between soybean rows two and three of each plot. After application, cards were collected and placed in the Zip Lock (S.C. Johnson & Son, Inc., Racine, WI, USA) storage bags. Cards were transferred to the laboratory where each was photographed using digital camera (Nikon D5600 18–58 mm NIKOR lens; Nikon Corporation, Tokyo, Japan) positioned 30 cm above the surface at a constant height across all samples. Images were then processed using Gotas computer software to estimate spray coverage.^34^, ^35^

**Table 1:** Site information and soil properties from soybean studies conducted in Wisconsin, USA (2023–2025)

Site, year	Crop information		Application dates	Soil properties	
	Hybrid	Planting date	POST	OM (%)	pH
Study 1. Spray coverage assessment – field environment					
Arlington, 2023	NK22‐C4E3^a^	9 May	19 June	3.5	6.7
Arlington, 2024	XO 2444E^b^	12 May	24 June	2.8	6.4
Study 3. Biological response of weeds to different sprayer settings – field environment					
Arlington, 2023	NK22‐C4E3^a^	9 May	19 June	3.5	6.7
Arlington, 2024	XO 2444E^b^	12 May	24 June	2.8	6.4
Janesville, 2023	NK22‐C4E3^a^	12 May	19 June	3.2	6.5
Janesville, 2024	XO 2444E^b^	15 May	21 June	3.3	6.7

*Note*: Seeding rate 345 800 seeds ha^−1^. Soil texture = Plano silt loam was distributed across both sites.

Abbreviations: OM, organic matter; POST, post‐emergence herbicide.

^a^ Syngenta Crop Protection.

^b^ Xitavo by BASF Agriculture.

**Table 2:** Application information, weather conditions, soybean growth stage, weed height, and weed density for all studies conducted in Wisconsin, USA (2023–2025)

	Temperature (^o^C)	RRH (%)	Cloud cover (%)	Wind speed (km h^−1^) and direction	Crop growth stage at application	Weed height (cm)	Weed density (m^2^)
Study 1. Spray coverage assessment							
Arlington, 2023	25	61	40	6 NE	V3/V4	14 ± 2	52 ± 14
Arlington, 2024	27	55	20	3 N	V3/V4	10 ± 3	50 ± 11
Study 2. Effect of wind on spray coverage							
Arlington, 2024	20	15	—	10 W	—	—	—
Study 3. Biological response of weeds to different sprayer settings							
Arlington, 2023	25	61	40	6 NE	V3/V4	14 ± 4	52 ± 14
Arlington, 2024	28	62	20	5 SE	V3/V4	15 ± 3	50 ± 11
Janesville, 2023	32	40	10	6 S	V3/V4	12 ± 2	44 ± 12
Janesville, 2024	25	79	10	5 SE	V3/V4	15 ± 4	50 ± 10
Study 4. Spray coverage to different nozzle fan angle							
Arlington, 2025	23	40	—	10 W	—	—	—
Study 5. Biological response of weeds to different nozzle orientation							
Brooklyn, 2025 A	30	88	0	1 E		7 ± 3	68 ± 12
Brooklyn, 2025 B	30	87	0	1.5 E		8 ± 2	55 ± 10

*Note*: Studies 2 and 4 were conducted at Arlington Agricultural Research Station, Arlington, WI, USA, in a closed shed. Weed density collected in the non‐treated check plots. Target weed in Arlington – common ragweed; target weed in Janesville – giant ragweed, Brookyln – waterhemp. Numbers after ‘±’ presented the standard error.

Abbreviation: RH, relative humidity.

### 2.2. Effect of wind on spray coverage (Study 2 – controlled environment)

**Figure 2:**
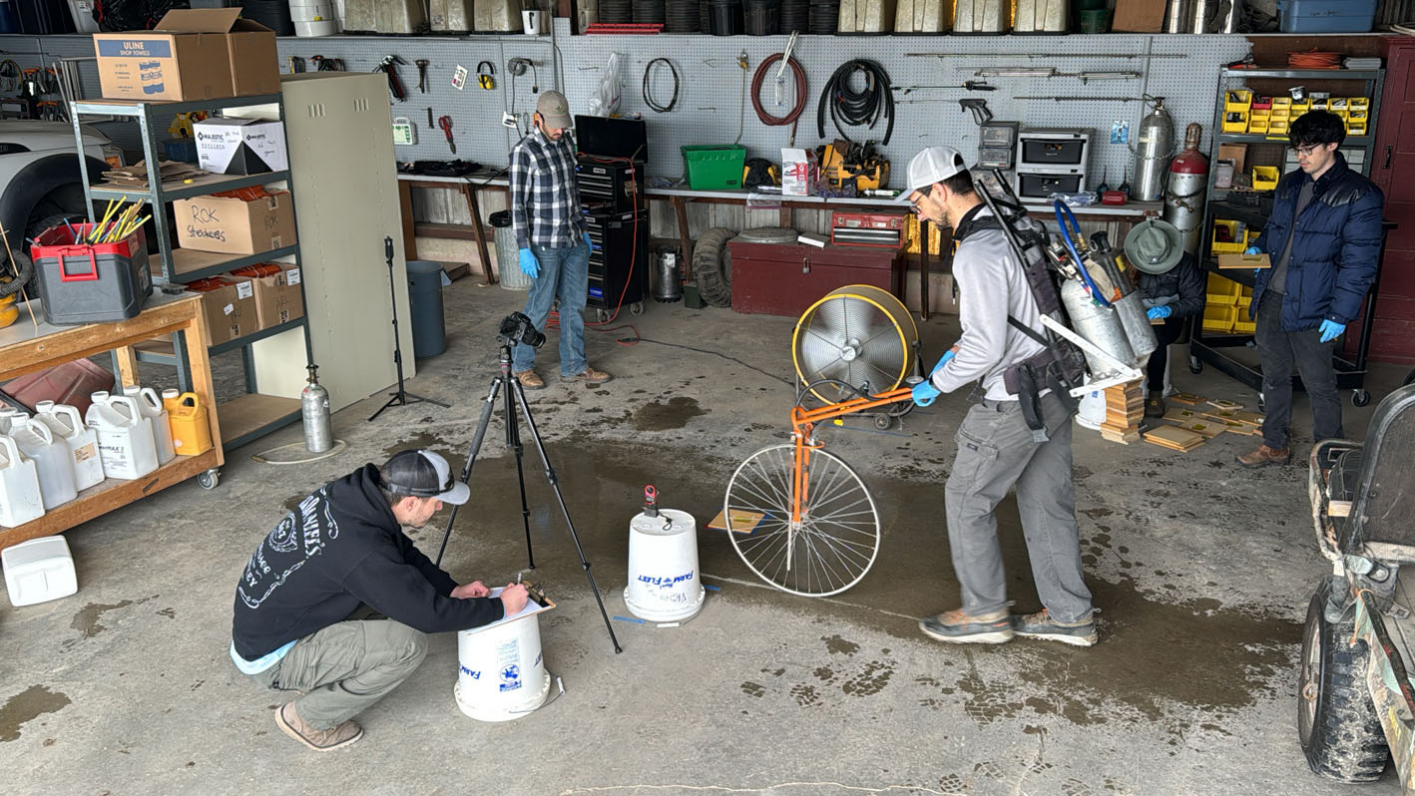
Controlled environment setup used to evaluate spray coverage and the influence of simulated wind during water application with a hand‐held boom.

Following spray pattern displacement observations during application in Study 1 (2023; Ugljic and Werle field observations), a subsequent study was conducted in an enclosed environment during the spring of 2024 at the Arlington Agricultural Research Station to investigate the impact of wind on spray coverage. The study was arranged as a factorial design in RCBD, with four replicates and two experimental runs. Treatments consisted of the same nozzle types, numbers of nozzles, and boom heights as mentioned in Study 1, with the additional factor of wind conditions (no wind and yes wind). A shop fan (Maxx Air, Mineral Wells, TX, USA) was used as the wind source which was positioned perpendicular (90°) to the spray pass (Fig. 2). Although airflow generated by a fan does not fully replicate natural field wind conditions, this setup was used to explore proof‐of‐concept responses under controlled airflow conditions. Anemometer (Kestrel 3000; Kestrel Instruments, Boothwyn, PA, USA) was placed 2.5 m from the fan measuring a crosswind speed. The wind speed was in the range from 9.6 to 11.5 km h^−1^, with an average wind speed of 10 km h^−1^ (Table 2). Water‐sensitive cards (7.6 cm × 5.2 cm; Syngenta) were placed 2 m from the fan on the wooden holder (10 cm × 10 cm) placed on flat soil surface, aligning with the middle nozzle of our three‐nozzle boom with nozzle spacing of 38 cm. Water‐sensitive cards were immediately collected, placed in Zip Lock storage bags, and transferred to a cooler to avoid the excess humidity from damaging samples.^50^ To prevent moisture interference affecting spray card integrity and data accuracy, wooden card holders were replaced with dry ones between treatments. Card spray coverage processing was conducted as described in Study 1.

### 2.3. Biological response of weeds to different sprayer settings (Study 3 – field environment)

A field study was conducted in 2023 and 2024 at two locations in Wisconsin (Table 1): Arlington Agricultural Research Station (43.30° N, 89.34° W) and the Rock County Farm in Janesville, WI, USA (42.72° N, 89.02° W) to evaluate weed control efficacy using a simulated THAT configured with different nozzle types, number of nozzles, and boom height treatments (Table 1). The study was arranged as a factorial design within RCBD, with four replications. The experimental unit size, soybean planting density, and treatments (nozzle types, number of nozzles used, and boom heights) were consistent with Study 1, with inclusion of non‐treated control (NTC) as a control treatment. The predominant weed species at Arlington was common ragweed (*Ambrosia artemisiifolia* L.), whereas giant ragweed (*Ambrosia trifida* L.) was the predominant species at Janesville. Weather conditions and application information are provided in Table 2. All treatments received glufosinate (Liberty 280 SL; BASF, Research Triangle, NC, USA) at postemergence (POST) at the rate of 656 g active ingredient (a.i.) ha^−1^ with 3.4 kg ha^−1^ of ammonium sulfate. Weed heights and soybean growth stage at the time of application are provided in Table 2. Visual weed control assessment on the scale 0 (no control) to 100% (complete control) compared to NTC was taken 14 days after treatment (DAT).^36^ Weed biomass samples were collected 14 DAT, where three sub‐samples were collected between soybean rows two and three of each plot (each sample was taken using 0.4 m^2^). Weed biomass within each quadrat was collected by clipping all aboveground vegetation at the soil surface. Samples were dried in a forced‐air oven at 54 °C for 1 week, or until a constant weight was achieved, and dry biomass was subsequently recorded.^36^, ^37^ Biomass data were converted to percent biomass reduction compared to NTC.^37^, ^38^

### 2.4. Spray coverage to different nozzle fan angle (Study 4 – controlled environment)

This controlled environment study was conducted in spring 2025 at the Arlington Agricultural Research Station to evaluate the impact of nozzle orientation (conventional 0° straight down *versus* 30° rearward inclined using QJ30 adapter by TeeJeet Spraying Systems Co.) on spray coverage with and without presence of wind. The study followed a factorial arrangement in a RCBD with four replicates and two experimental runs. Treatments included two boom heights (53 cm *versus* 76 cm), nozzle orientation (conventional 0° *versus* 30° rearward inclined) and wind conditions as described in Study 2. Hand boom mounted on a bicycle wheel equipped with three DG80015 nozzles (TeeJet Spraying Systems Co.) was calibrated as described in Study 1. Water‐sensitive cards were used following the same procedures and data collection methods described in Study 2.

### 2.5. Biological response of weeds to different nozzle fan angle (Study 5 – field environment)

Bareground field studies were conducted during summer 2025 at O’Brien Family Farm near Brooklyn, WI, USA, to evaluate the impact of nozzle orientation (conventional 0° straight down *versus* 30° rearward inclined) on weed control. The studies followed a factorial arrangement in a RCBD with four replicates and two experimental runs. Treatments included two boom heights (53 cm *versus* 76 cm), nozzle orientation (conventional 0° *versus* 30° rearward inclined) and NTC treatment. Hand boom mounted on a bicycle wheel equipped with three DG80015 nozzles (TeeJet Spraying Systems Co.) was calibrated as described in Study 1. Weather conditions, weed demographics, and application details are summarized in Table 2. Predominant weed species was waterhemp (*Amaranthus tuberculatus* (Moq.) Sauer). All treatments received glufosinate as described in Study 3. Visual weed control and biomass reduction were evaluated 14 DAT following the methods described in Study 3.

### 2.6. Data analysis

All data were analyzed using RStudio statistical software^39^ (version 2025.05.1+513). Nozzle type, number of nozzles, nozzle orientation, and boom height were considered as the main effects. Spray coverage data from Studies 1, 2, and 4 were analyzed with replications nested within site‐years or experimental runs, which were treated as random effects, since no significant interactions were detected between site‐years. In Study 3, weed control and biomass reduction data were analyzed separately for each location (Arlington and Janesville) due to differences in weed species, and replications were nested within years which were treated as random effects; biomass was measured from three sub‐samples per plot, which were averaged prior to analysis and treated as a single value for each replication. In Study 5, weed control and biomass from the field study were analyzed across both site‐years, with replication nested within site‐years and treated as a random effect. All data were analyzed using generalized linear mixed models fitted by maximum likelihood with a Laplace approximation using the *glmmTMB* package.^40^ Data from all studies were analyzed using beta distribution (link = logit) on scale 0 to 1 to meet model assumptions (*glmmTMB* package)^40^ and back‐transformed data are presented for ease of interpretation.^23^, ^41^ Treatment means were separated using Tukey's honest significant difference at *ɑ* = 0.05 using *emmeans* package.^42^

## 3. RESULTS

### 3.1. Spray coverage assessment (Study 1)

The two‐way interaction nozzle type × number of nozzles and the main effect of boom height influenced spray coverage (Table 3 and Supporting Information Table S1). Multiple TP40015E nozzles provided better spray coverage (43%) than multiple DG80015 nozzles (37%). A single TP40015E nozzle provided better spray coverage (29%) than DG80015 nozzle which provided the least spray coverage (16%). Boom height of 53 cm provided greater spray coverage (34%) compared to 76 cm boom height (28%).

**Table 3:** The effect of nozzle type, number of nozzles and boom height on spray coverage in soybean studies conducted in Arlington and Janesville, WI, USA, in 2023 and 2024 (Study 1)

		Spray coverage (%)	
Nozzle type			
	TP40015E	36	a
	DG80015	26	b
*P* Value < 0.0001			
Number of nozzles			
	Single	22	b
	Multiple	40	a
*P* Value < 0.0001			
Boom height			
	53 cm	34	a
	76 cm	28	b
*P* Value = 0.0022			
Nozzle type × Number of nozzles			
Nozzle type	Number of nozzles		
TP40015E	Single	29	c
TP40015E	Multiple	43	a
DG80015	Single	16	d
DG80015	Multiple	37	b
*P* Value = 0.0003			
Nozzle type × Boom height			
Nozzle type	Boom height (cm)		
TP40015E	53	37	
TP40015E	76	34	
DG80015	53	30	
DG80015	76	23	
*P* Value = 0.1298			
Number of nozzles × Boom height			
Number of nozzles	Boom height (cm)		
Single	53	26	
Single	76	19	
Multiple	53	42	
Multiple	76	38	
*P* Value = 0.0518			

*Note*: Letters indicate group differences between means of treatments (Tukey's honest significant difference, *α* = 0.05).

### 3.2. Effect of wind on spray coverage (Study 2)

Two‐way interactions of nozzle type × number of nozzles, nozzle type × wind, number of nozzles × wind, and boom height × wind had significant impact on spray coverage (Tables 4 and S2). Multiple nozzles, regardless of their type, provided greatest spray coverage (42–43%), and the use of a single TP40015E nozzle provided better spray coverage (32%) than DG80015 nozzle, which provided the lowest spray coverage in this study (23%). TP40015E nozzle provided greatest spray coverage (51%) in no wind condition, while in presence of wind, both nozzle types (TP40015E and DG80015) provided similar levels of spray coverage (25%). Multiple nozzles provided the greatest spray coverage in no wind conditions (58%) whereas the lowest spray coverage (19%) was observed with a single nozzle in presence of wind. The boom height × wind interaction indicated that in the presence of wind, increasing boom height substantially reduced spray coverage, with the lowest coverage (18%) observed at 76 cm using a single nozzle.

**Table 4:** The effect of nozzle type, number of nozzles, boom height and wind on spray coverage in controlled environment conducted in Arlington, Wisconsin in 2024 (Study 2)

		Spray coverage (%)	
Nozzle type × number of nozzles			
Nozzle type	Number of nozzles		
TP40015E	Single	32	b
TP40015E	Multiple	42	a
DG80015	Single	23	c
DG80015	Multiple	43	a
*P* Value = 0.0054			
Nozzle type × wind			
Nozzle type	Wind		
TP40015E	No	51	a
DG80015	No	42	b
TP40015E	Yes	25	c
DG80015	Yes	25	c
*P* Value = 0.0179			
Nozzle type × boom height			
Nozzle type	Boom height (cm)		
TP40015E	53	42	
TP40015E	76	31	
DG80015	53	41	
DG80015	76	26	
*P* Value = 0.1996			
Number of nozzles × wind			
Number of nozzles	Wind		
Single	No	36	b
Multiple	No	58	a
Single	Yes	19	d
Multiple	Yes	29	c
*P* Value = 0.0011			
Number of nozzles × Boom height			
Number of nozzles	Boom height (cm)		
Single	53	36	
Single	76	21	
Multiple	53	48	
Multiple	76	38	
*P* Value = 0.5574			
Boom height × wind			
Boom height (cm)	Wind		
53	No	52	a
76	No	41	b
53	Yes	32	c
76	Yes	18	d
*P* Value = 0.0374			

*Note*: Letters indicate group differences between means of treatments (Tukey's honest significant difference, *α* = 0.05).

### 3.3. Biological response of weeds to different sprayer settings (Study 3)

#### 3.3.1. Common ragweed visual weed control assessment and biomass reduction

The effect of the number of nozzles influenced visual common ragweed control assessment, whereas number of nozzles and boom height effected common ragweed biomass reduction (Tables 5 and S3). The use of multiple nozzles provided better weed control (92%) and biomass reduction (96%) compared to a single nozzle (78% weed control and 87% biomass reduction). Boom height of 53 cm provided greatest biomass reduction (94%) compared to 76 cm boom height treatments (88%).

**Table 5:** The effect of nozzle type, number of nozzles, boom height on weed control and biomass reduction 14 days after treatment in Arlington (common ragweed) and Janesville (giant ragweed), WI, USA, in 2023 and 2024 (Study 3)

		Weed control (%)	Biomass reduction (%)
Arlington 2023–2024 common ragweed			
Nozzle type	TP40015E	87	92
	DG80015	83	91
	*P* Value	0.2799	0.5546
Number of nozzles	Single	78 b	87 b
	Multiple	92 a	96 a
	*P* Value	<0.0001	<0.0001
Boom height (cm)	53	87	94 a
	76	82	88 b
	*P* Value	0.1070	0.02979
Janesville 2023–2024 giant ragweed			
Nozzle type	TP40015E	93	91
	DG80015	91	88
	*P* Value	0.3035	0.5553
Number of nozzles	Single	88 b	83 b
	Multiple	96 a	95 a
	*P* Value	0.0027	0.0107
Boom height (cm)	53	95 a	95 a
	76	88 b	84 b
	*P* Value	0.05003	0.0189

*Note*: Letters indicate group differences between means of treatments (Tukey's honest significant difference, *α* = 0.05).

#### 3.3.2. Giant ragweed visual weed control assessment and biomass reduction

The effect of number of nozzles and boom height influenced both visual weed control assessments and biomass reduction (Tables 5 and S3). The use of multiple nozzles resulted in better weed control of giant ragweed (96%) than a single nozzle (88%) and provided more biomass reduction (95%) than a single nozzle (83%). A boom height of 53 cm resulted in greatest weed control (95%) and biomass reduction (95%) compared to the 76 cm boom height, which achieved 88% weed control and 84% biomass reduction.

### 3.4. Spray coverage to different nozzle orientation (Study 4)

The two‐way interactions of boom height × wind and nozzle orientation × wind influenced spray coverage (Tables 6 and S4). Boom height of 53 cm provided the greatest spray coverage (45%) in no wind condition, while lowest spray coverage (14%) was observed at 76 cm in presence of wind. Both nozzle orientations (conventional 0° and 30° rearward inclined) in no wind conditions provided similar spray coverage (42%), while lowest spray coverage (16%) was observed with 30° rearward inclined nozzles in presence of wind. Wind influenced spray coverage, with 43% coverage under no wind conditions compared to 19% in presence of wind.

**Table 6:** The effect of nozzle angles, boom height and wind on spray coverage in controlled environment conducted in Arlington, WI, USA, in 2025 (Study 4)

		Spray coverage (%)	
Boom × wind			
Boom height (cm)	Wind		
53	No	45	a
76	No	41	b
53	Yes	25	c
76	Yes	14	d
*P* Value ≤ 0.0001			
Nozzle angle × wind			
Nozzle angle (deg)	Wind		
0	No	42	a
30	No	43	a
0	Yes	22	b
30	Yes	16	c
*P* Value ≤ 0.0001			

*Note*: Letters indicate group differences between means of treatments (Tukey's honest significant difference, *α* = 0.05).

### 3.5. Biological response of weeds to different nozzle orientation (Study 5)

The effect of nozzle orientation and boom height did not influence visual weed control assessment and biomass reduction in this field study (Tables 7, S5 and S6). All treatments provided > 97% waterhemp control and 99% biomass reduction.

**Table 7:** The effect of nozzle angles and boom height on weed control and biomass reduction in field environment conducted in Brooklyn, WI, USA, in 2025 (Study 5).

		Weed control (%)	Biomass reduction (%)
Nozzle angle (deg)	0	97	99
	30	98	99
*P* Value		0.6051	0.6064
Boom height (cm)	53	98	99
	76	98	99
*P* Value		0.1473	0.4425

*Note*: Letters indicate group differences between means of treatments (Tukey's honest significant difference, *α* = 0.05). Predominant weed species in this study was waterhemp.

## 4. DISCUSSIONS

It is well documented that spray solution characteristics (e.g., product formulation, viscosity), nozzle types, droplet size, boom height, carrier volume, and environmental conditions influence spray coverage, which in turn can affect weed control efficacy.^25^, ^26^, ^27^, ^28^, ^43^ Contact herbicides such as glufosinate, are more dependent on adequate spray coverage than systemic herbicides like glyphosate to provide effective control of susceptible species.^20^, ^38^, ^44^, ^45^ Our results were consistent with previous studies, demonstrating that under field conditions, weed control greater than 88% is achievable with both even and overlapping nozzles. We observed that both nozzle types provided similar levels of weed control and spray coverage under no wind conditions. However, the use of a single even nozzle, particularly in the presence of wind and boom sway during application, may result in insufficient spray coverage, sub‐label herbicide rates, and spray swath displacement/off‐target movement.^24^, ^30^, ^46^

In our study, the use of multiple nozzles resulted in better spray coverage (40%), weed control (92%), and biomass reduction (96%) compared to a single nozzle, regardless of nozzle type or boom height. Moreover, activating multiple nozzles can improve control of weeds that are not detected by the camera system but are still treated by chance through overlapping spray patterns, in contrast to using a single narrow‐angle nozzle,^16^ provided that weeds are not shielded by larger plants or intercepted by the crop canopy. Alheidary *et al*. reported that when multiple nozzles were activated, adjacent nozzles under crosswind conditions reduced spray swath displacement and maintained better spray deposition on the targeted area.^46^ Our results support the use of multiple nozzles to improve spray coverage and ensure effective weed control with THATs, especially in the presence of wind when spray swath displacement is likely. Similar outcomes were reported by Villette *et al*., they demonstrated through computer simulations that increased nozzle overlap improves spray uniformity and adherence to target application rates, key factors for effective weed management.^24^ While their study was based on modeling, our results provide experimental confirmation that activating multiple nozzles upon weed detection improves spray coverage and contributes to more effective weed control compared to single nozzle activation. Although the dose delivered to individual plants was not directly quantified in this study, the selected boom height resulted in a TP nozzle spray band width approximately equal to the nozzle spacing, which likely explains the similar performance of TP nozzles under single and multiple nozzle activation. In contrast, the DG nozzle produced a wider spray band, leading to effective dose dilution when operated as a single nozzle, thereby highlighting the value of multiple‐nozzle activation for improving spray coverage and reducing the risk of sub‐lethal herbicide doses when using overlapping nozzles. Conversely, excessive nozzle overlap, particularly when even flat‐fan nozzles (TP40015E) are operated either above or below the ideal boom height (53 cm) relative to nozzle spacing (38 cm) can result in localized over‐dosing and the potential to exceed maximum allowable label doses. Therefore, careful optimization of boom height and nozzle selection is essential to ensure adequate spray coverage while avoiding either under‐ and over‐dosing.

Nozzle orientation influenced spray coverage in the controlled environment study (Study 4) but did not result in differences in weed control or biomass reduction in the field experiment (Study 5). This was anticipated, as field applications in this study were conducted under no wind conditions (Table 2), which provided similar spray coverage across nozzle orientations and boom heights. Therefore, under no wind conditions, nozzle orientation did not influence weed control. Consistent with this, Vitti^30^ reported that nozzles oriented 30° rearward inclined resulted in lower spray coverage, particularly at increased boom heights. However, further studies are needed to assess the effects of nozzle orientation under varying nozzle types, travel speeds, and environmental conditions, such as wind speed and direction, which may affect spray deposition and herbicide efficacy. From a THAT perspective, rearward inclined nozzles offer advantages for real‐time weed detection, as the processing unit has more time to identify weeds and potentially enabling higher travel speed.^21^ Future research should determine whether this configuration could allow higher travel speeds without compromising weed control.

Boom height plays a crucial role in minimizing drift, achieving desired spray coverage and weed control.^47^ Stallinga *et al*. documented that lower boom heights (50 cm *versus* 70 cm) reduced spray drift by up to 50%, thereby improving herbicide deposition on target.^48^ Our findings further support that a 53 cm boom height provided better spray coverage under both no‐wind and wind conditions (Study 2) and resulted in greater weed control and biomass reduction compared with a 76 cm boom height (Study 3). While reduced boom height has clear benefits, excessively low boom height can compromise spray uniformity and increase the risk of boom damage due to contact with the ground caused by vertical boom sway/movement.^48^, ^49^ Moreover, operating a sprayer at low boom heights may require a narrower nozzle spacing to maintain spray volume, potentially leading to uneven spray coverage^48^, ^49^ which can lead to high under spraying or streaking using even nozzles. The findings of this study underscore the importance of selecting an optimal boom height and maintaining boom stability to achieve consistent and effective herbicide application. In practice, applicators may operate at sub‐optimal taller heights to prevent wide booms from contacting the ground, and even when the ideal height is set, boom instability, caused by uneven terrain or excessive travel speed, can lead to uneven spray deposition. Additionally, avoiding applications during windy conditions is essential to minimize spray swath displacement and off‐target movement, as our results showed reduced spray coverage in presence of wind, which can be especially detrimental in targeted application systems. Although this study did not evaluate boom sway mitigation strategies, approaches such as reducing travel speed, or incorporating technologies (e.g., boom height sensors) or mechanical aids (e.g., boom‐following wheels, carbon fiber booms), maintaining consistent boom height across the field remains warranted and should be considered in future research and equipment development.

## 5. CONCLUSIONS

This study demonstrated that nozzle type, number of activated nozzles, nozzle orientation, boom height, and wind conditions can significantly affect spray coverage and subsequent weed control efficacy of THATs. Activating multiple nozzles consistently improved spray coverage, weed control and biomass reduction compared to single nozzle activation, regardless of nozzle type or boom height. The recommended boom height, 53 cm for the nozzle types and spacing used in these experiments, provided greater spray coverage (Study 1), particularly in presence of wind (Study 2), and weed control (Study 3). Either nozzle orientation (conventional 0° *versus* 30° rearward inclined) provided similar spray coverage and weed control in no wind condition. However, in the presence of wind, 30° rearward inclined nozzle at 76 cm boom height provided the lowest spray coverage. These findings confirm previous modeling‐based predictions on the value of overlapping nozzles and highlight the operational importance of boom stability for optimizing THATs. Until single even nozzle strategies are refined for consistent performance under variable field and environmental conditions, multiple overlapping nozzle activation remains a more reliable approach for effective weed management using THATs.

## CONFLICT OF INTEREST

No conflicts of interest have been declared.

## Supporting information


**Figure S1.** Schematic representation of theoretical spray coverage used in this study. Theoretical spray width (*W*) was calculated as a function of boom height (*H*) and spray angle (*θ*) using the equation *W* = 2 × *H* × tan(*θ*/2), where *W* represents theoretical spray width (cm), *H* represents spray distance or boom height (cm), and *θ* represents nozzle spray angle (degrees). An example calculation is shown for a boom height of 53 cm and a spray angle of 40°.

**Table S1.** Summary of analysis of variances of main effects and interactions on spray coverage, Study 1 – Field Conditions at Arlington, Wisconsin in 2023 and 2024. Data was pooled across 2 years.
**Table S2.** Summary of analysis of variances of main effects and interactions on spray coverage Study 2 in controlled environment with and without presence of wind at Arlington, Wisconsin in 2024. Data pooled across two experimental runs.
**Table S3.** Summary of analysis of variances of main effects and interactions on weed control and biomass reduction, Study 3, field studies at Arlington and Janesville, Wisconsin in 2023 and 2024. Data was pooled across years.
**Table S4.** Summary of analysis of variances of main effects and interactions on spray coverage, Study 4 – Controlled environment at Arlington, Wisconsin in 2025. Data was pooled across two experimental runs.
**Table S5.** Summary of analysis of variances of main effects and interactions on weed control, Study 5 – Field study at Brooklyn, Wisconsin in 2025.
**Table S6.** Summary of analysis of variances of main effects and interactions on biomass reduction, Study 5 – Field study at Brooklyn, Wisconsin in 2025.

## Data Availability

The data that support the findings of this study are available from the corresponding author upon reasonable request.
