# Supplementary material for: Targeted herbicide spraying systems: role of nozzle type, number of nozzle activation, nozzle orientation, and boom height on spray coverage and weed control
Source: Pest Manag Sci. 2026 Feb 21;82(6):5216–24. doi: 10.1002/ps.70629 (PMC13158438; doi:10.1002/ps.70629)
Supplement: Supplementary file 1 — Figure S1. Schematic representation of theoretical spray coverage used in this study. Theoretical spray width (W) was calculated as a function of boom height (H) and spray angle (θ) using the equation W = 2 × H × tan(θ/2), where W represents theoretical spray width (cm), H represents spray distance or boom height (cm), and θ represents nozzle spray angle (degrees). An example calculation is shown for a boom height of 53 cm and a spray angle of 40°. [file PS-82-5216-s002.pdf]

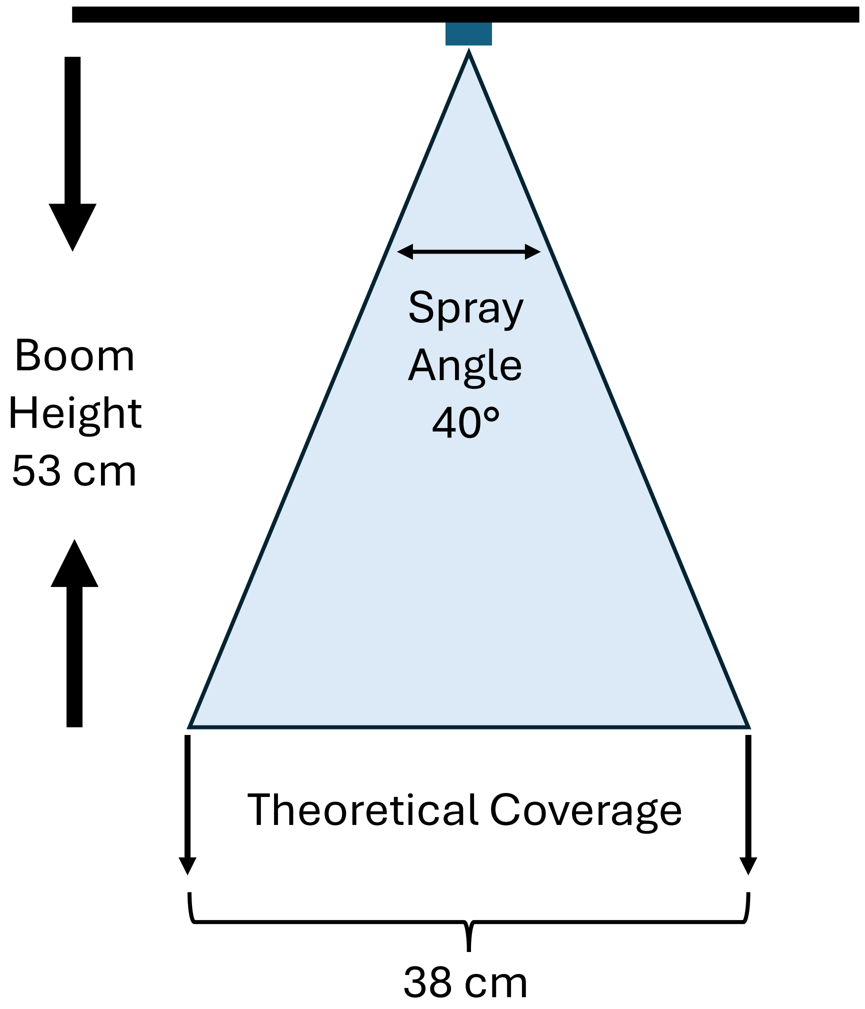

Equation: W = 2 x H x tan (θ / 2)

W - Theoretical spray width (cm)
H – Spray distance (cm)
Θ – Spray angle (degrees)
