# Supplementary material for: Targeted herbicide spraying systems: role of nozzle type, number of nozzle activation, nozzle orientation, and boom height on spray coverage and weed control
Source: Pest Manag Sci. 2026 Feb 21;82(6):5216–24. doi: 10.1002/ps.70629 (PMC13158438; doi:10.1002/ps.70629)
Supplement: Supplementary file 2 — Table S1. Summary of analysis of variances of main effects and interactions on spray coverage, Study 1 – Field Conditions at Arlington, Wisconsin in 2023 and 2024. Data was pooled across 2 years. Table S2. Summary of analysis of variances of main effects and interactions on spray coverage Study 2 in controlled environment with and without presence of wind at Arlington, Wisconsin in 2024. Data pooled across two experimental runs. Table S3. Summary of analysis of variances of main effects and interactions on weed control and biomass reduction, Study 3, field studies at Arlington and Janesville, Wisconsin in 2023 and 2024. Data was pooled across years. Table S4. Summary of analysis of variances of main effects and interactions on spray coverage, Study 4 – Controlled environment at Arlington, Wisconsin in 2025. Data was pooled across two experimental runs. Table S5. Summary of analysis of variances of main effects and interactions on weed control, Study 5 – Field study at Brooklyn, Wisconsin in 2025. Table S6. Summary of analysis of variances of main effects and interactions on biomass reduction, Study 5 – Field study at Brooklyn, Wisconsin in 2025. [file PS-82-5216-s001.docx]

| **Supplemental. Table 1.** Summary of analysis of variances of main effects and interactions on spray coverage, Study 1- Field Conditions at Arlington, Wisconsin in 2023 and 2024. Data was pooled across two years. | |
| --- | --- |
|  | Spray coverage |
| **Anova** | P value |
| Nozzle type | <0.0001 |
| Number of nozzles | <0.0001 |
| Boom height | 0.0022 |
| Nozzle type × number of nozzles | 0.0003 |
| Nozzle type × boom height | 0.1298 |
| Number of nozzles × boom height | 0.0518 |
| Nozzle type × number of nozzles × boom height | 0.0741 |
|  | |

| **Supplemental. Table 2.** Summary of analysis of variances of main effects and interactions on spray coverage Study 2 in controlled environment with and without presence of wind at Arlington, Wisconsin in 2024. Data pooled across two experimental runs. | |
| --- | --- |
| **Study 2** | Spray coverage |
| **Anova** | P value |
| Nozzle type | <0.0001 |
| Number of nozzles | <0.0001 |
| Boom height | <0.0001 |
| Wind | <0.0001 |
| Nozzle type × number of nozzles | 0.0054 |
| Nozzle type × boom height | 0.2554 |
| Nozzle type × wind | 0.0179 |
| Number of nozzles × boom height | 0.0258 |
| Number of nozzles × wind | 0.0011 |
| Boom height× wind | 0.0374 |
| Nozzle type × number of nozzles × boom height | 0.5868 |
| Nozzle type × number of nozzles × wind | 0.1807 |
| Nozzle type × boom height × wind | 0.3642 |
| Number of nozzles × boom height × wind | 0.8066 |
| Nozzle type × number of nozzles × boom height × wind | 0.4758 |
|  | |

| **Supplemental. Table 3.** Summary of analysis of variances of main effects and interactions on weed control and biomass reduction, Study 3, field studies at Arlington and Janesville, Wisconsin in 2023 and 2024. Data was pooled across years. | | | | |
| --- | --- | --- | --- | --- |
|  | Weed control 14 DAT | | Biomass reduction | |
|  | Common ragweed | Giant ragweed | Common ragweed | Giant ragweed |
| Nozzle type | 0.0398 | 0.1452 | 0.0218 | 0.8498 |
| Number of nozzles | <0.0001 | 0.0030 | <0.0001 | 0.0176 |
| Boom height | 0.0283 | 0.0061 | 0.0021 | 0.0077 |
| Nozzle type × number of nozzles | 0.3389 | 0.0617 | 0.1247 | 0.0695 |
| Nozzle type × boom heigh | 0.8998 | 0.2352 | 0.2319 | 0.8048 |
| Number of nozzles × boom height | 0.2736 | 0.4742 | 0.1766 | 0.7563 |
| Nozzle type × number of nozzles × boom height | 0.8797 | 0.3419 | 0.5554 | 0.8220 |
|  | | | | |

| **Supplemental. Table 4.** Summary of analysis of variances of main effects and interactions on spray coverage, Study 4- Controlled environment at Arlington, Wisconsin in 2025. Data was pooled across two experimental runs. | |
| --- | --- |
|  | Spray coverage |
|  | P value **-** |
| Nozzle angle | 0.3378 |
| Boom height | 0.5250 |
| Wind | <0.0001 |
| Boom height × nozzle angle | 0.0796 |
| Boom height × wind | <0.0001 |
| Nozzle angle × wind | <0.0001 |
| Boom height × nozzle angle × wind | 0.0624 |
|  | |

| **Supplemental. Table 5.** Summary of analysis of variances of main effects and interactions on weed control, Study 5- Field study at Brooklyn, Wisconsin in 2025. | |
| --- | --- |
|  | Weed control |
|  | P value |
| Nozzle angle | 0.6051 |
| Boom height | 0.1473 |
| Nozzle angle × boom height | 0.4321 |
|  | |

| **Supplemental. Table 6.** Summary of analysis of variances of main effects and interactions on biomass reduction, Study 5- Field study at Brooklyn, Wisconsin in 2025. | |
| --- | --- |
|  | Biomass reduction |
|  | P value |
| Nozzle angle | 0.6456 |
| Boom height | 0.4425 |
| Nozzle angle × boom height | 0.8658 |
|  | |
